# The influence of the respiratory cycle on reaction times in sensory-cognitive paradigms

**DOI:** 10.1038/s41598-022-06364-8

**Published:** 2022-02-16

**Authors:** Michelle Johannknecht, Christoph Kayser

**Affiliations:** 1 Institute of Clinical Neuroscience and Medical Psychology, Medical Faculty, Heinrich-Heine-University Düsseldorf, Düsseldorf, Germany; 2 Department for Cognitive Neuroscience, Faculty of Biology, Bielefeld University, Bielefeld, Germany

**Keywords:** Cognitive neuroscience, Perception, Neuroscience

## Abstract

Behavioural and electrophysiological studies point to an apparent influence of the state of respiration, i.e., whether we inhale or exhale, on brain activity and cognitive performance. Still, the prevalence and relevance of such respiratory-behavioural relations in typical sensory-cognitive tasks remain unclear. We here used a battery of six tasks probing sensory detection, discrimination and short-term memory to address the questions of whether and by how much behaviour covaries with the respiratory cycle. Our results show that participants tend to align their respiratory cycle to the experimental paradigm, in that they tend to inhale around stimulus presentation and exhale when submitting their responses. Furthermore, their reaction times, but not so much their response accuracy, consistently and significantly covary with the respiratory cycle, differing between inhalation and exhalation. This effect is strongest when analysed contingent on the respiratory state around participants’ responses. The respective effect sizes of these respiration-behaviour relations are comparable to those seen in other typical experimental manipulations in sensory-cognitive tasks, highlighting the relevance of these effects. Overall, our results support a prominent relation between respiration and sensory-cognitive function and show that sensation is intricately linked to rhythmic bodily or interoceptive functions.

## Introduction

Breathing is a vital and automatic process that can be consciously exploited to adapt to behavioural challenges or to control our bodily and mental state. The brain structures controlling breathing and those sensing the resulting changes in airflow are intricately connected with the limbic system^1^. Thereby, information about the respiratory state is potentially widely available in subcortical and cortical brain regions^2^. This gives us the potential to consciously adjust breathing such as during singing but may also allow respiratory-related signals to continuously and subconsciously influence perceptual or cognitive functions^3–6^.

In line with the notion that respiration may influence neocortical function, activity in multiple brain regions apparently co-modulates with the respiratory cycle, possibly reflecting the propagation of feedback signals about inhalation or exhalation (further called the respiratory state) for use in sensory-cognitive processes^3,7–9^. Behavioural studies have shown that animal or human participant’s performance can covary with the respiratory cycle in a variety of tasks, ranging from sensory detection^10,11^ to emotion recognition^9,12–14^, memory recall^9,15–17^, or more complex mental tasks^7^. Also, in motor tasks participants preferentially or more swiftly trigger actions during specific respiratory states^18,19^. Often these effects are stronger for nasal than oral breathing, in line with olfactory structures prominently providing feedback about respiratory action to cortical regions^9^﻿.

While these previous studies collectively suggest that perceptual judgements indeed vary between inhalation and exhalation, the prevalence and relevance of such effects during typical laboratory tasks remains unclear. First, previous studies used diverse methods for registering participants respiratory state (respiratory belts, pneumotachographs, manometers) and different analytical approaches to detect the potential covariation of respiration and behaviour. This makes it difficult to compare results across behavioural assays and to establish effect sizes of the behaviour-respiratory relation. Second, many studies explicitly instructed participants to a specific type of respiration (oral, nasal, deep respiration), which may bias participant’s attention to their own respiration and may amplify potential effects^6,8,14^. Furthermore, typical laboratory paradigms are often structured around specific event times, such as stimulus onsets or participant’s responses. Previous work has not investigated whether the respiratory state during sensory onsets or around the time of participants’ responses more strongly relates to behavioural performance.

The present study was designed to probe whether and by how much participant’s performance in typical perceptual and memory tasks varies along the respiratory cycle. For this we exploited a sensitive measurement of respiratory airflow^20,21^ and quantified the variation of response accuracy and reaction times along the respiratory cycle of human participants in six tasks. Importantly, participants performed these tasks without a specific constraint on how to breathe and were simply instructed to “breathe through their nose as usual”. This instruction was used to mimic typical ‘every-day’ experiments that do not impose specific manipulations on participant’s respiration. The tasks were either based on previous studies reporting a relation between memory performance or judgments of emotions and respiration^9^ or were auditory and visual detection or discrimination tasks employed in our previous work. We analysed the data both when combined across all tasks and for each task individually, probing two main questions: whether participants tend to align their respiratory behaviour to the experimental task, and whether performance (reaction time, response accuracy) systematically covaries with the respiratory cycle.

## Methods

### Participants

A total of 42 adult volunteers participated in this study. The procedures were in accordance with the Declaration of Helsinki and were approved by the ethics committee of Bielefeld University. Participants gave written informed consent and were informed about the general procedures and the nature of the individual tasks prior to participating. Our specific interest to investigate the relation between respiration and task performance was not mentioned explicitly prior to the study. A total of six paradigms was used and the data for these were collected in two independent groups of participants. We initially aimed to obtain at least 20 valid datasets for each paradigm, based on general recommendations for behavioural studies^22^. However, this goal was not reached for one paradigm (see below). In practice, we collected data from 21 participants in group 1 (emotion discrimination, visual memory, and sound detection; 12 females, mean age 25.5 ± 3.3 years), and from 21 participants in group 2 (two pitch discrimination and a visual motion task; 13 females, mean age 24.2 ± 3 years). Some individual datasets had to be excluded, as described below. Participants received 10€ per hour as compensation for their time.

### General procedures

The experiments were performed in a darkened and sound-proof booth (E: Box; Desone, Germany). Participants sat comfortably in front of a monitor (27″ monitor; ASUS PG279Q, 120 Hz refresh rate, grey background of 16 cd/m^2^) and two speakers were positioned adjacent to the left and right of the monitor. Stimulus presentation was controlled using the Psychophysics Toolbox (Version 3.0.14; http://psychtoolbox.org/) using MATLAB (Version R2017a; The MathWorks, Inc., Natick, MA) and was synchronized to an EEG recording system using TTL pulses. Participants responded using a computer keyboard. We also monitored eye movements using an EyeLink 1000 System (monitoring their right eye at 250 Hz). However, this was technically not possible for some of the participants and the eye tracking data were not analysed for this study.

### Recording of respiratory signals

During the main experiments participants wore disposable clinical oxygen masks from which the respiratory-tube connectors were removed. In the opening of these connecters a temperature- sensitive resistor was inserted (Littelfuse Thermistor No. GT102B1K, Mouser electronics). This thermistor allows recording the continuous temperature changes resulting from the respiration-related airflow at high temporal resolution^20,21^. The continuous voltage drop across the thermistor was amplified using a custom-made circuit and recorded and digitized via the analogue input of an ActiveTwo EEG system (BioSemi BV,Netherlands) at a temporal resolution of 500 Hz. Participants were instructed to breathe normally through the nose, as if performing the experiments without wearing the mask. During the experiment we could not continuously monitor whether participants adhered to this instruction, but in a debriefing questionnaire all participants indicated that they breathed via their nose. Still, this leaves the possibility that during parts of the experiment participants were breathing orally.

### Behavioural paradigms

The following six behavioural paradigms were used. These were administered in two separate groups: Group 1 performed two pitch discrimination tasks (referred to as Pitch1 and Pitch 2 in the following) and a visual motion task (Motion) in counterbalanced order between participants. Group 2 performed an emotion discrimination task (Emotion), a visual memory task (Memory), and sound detection task (Sound), the order of which was the same across participants to ensure the same delay period for the memory part: first was the encoding session for the memory task, then emotion discrimination, then memory retrieval, and finally the sound detection task. In the following we describe each paradigm in detail. Trials started with a fixation period, which was the same for all paradigms (400–1000 ms, uniform) unless stated. Inter-trial intervals were the same for all paradigms (1200–1500 ms, uniform). Respiratory data was only collected during the main experiments but not during practice blocks or blocks used to determine psychometric thresholds. For each task, participants were instructed to respond as fast and accurately as possible after stimulus presentation.

### Pitch discrimination tasks

The pitch discrimination tasks involved judgements of the pitch of two brief successive tones, as used in two previous studies^23^. During each trial two pure tones (50 ms duration, 6 ms cosine ramp, 50 ms pause in between, 65 dB SPL) were presented and participants had two indicate which of the two (first, or second) had higher pitch. One tone was always a standard (1024 Hz) the other tone varied around this pitch in five octave-spaced levels. These levels were multiples of the participant-specific threshold, which was obtained separately ([0, 0.5, 1, 1.5, 2] * threshold). Trials started with a fixation period, following which the stimuli were presented. The paradigm consisted of three parts: a brief training session, a block to estimate participant’s thresholds, and finally the main task. Participant-specific thresholds were obtained using three-interleaved one-up two-down staircases with multiplicative step-sizes (starting at differences of 0.5, 0.1 and 0.02 octaves respectively). The three individual thresholds were averaged to yield the participant-specific threshold. The tasks Pitch1 and Pitch 2 differed in that during Pitch 1 the stimulus was presented following the random fixation period. During Pitch 2, participants had to manually initialize the start of the trial by pressing a key, which was then followed by a shorter fixation period (300 to 600 ms uniform). For Pitch 1 participants performed two blocks of 200 trials each (resulting in 80 repeats per pitch difference), for Pitch 2 participants performed one block of 200 trials (resulting in 40 repeats per difference). For both tasks the data from all n = 21 participants could be analysed.

### Visual motion task

In this task, participants judged the direction of motion (left- or right-wards) of visual random dot displays. Random dot displays lasted 340 ms, subtended 10 degrees of visual angle and contained 1100 limited-lifetime dots (0.2° diameter, 8 frames life-time) moving at 5 degrees per second. The coherence of the dots (fraction of dots moving in the same direction) varied across five levels around participant’s individual thresholds ([0.55, 0.77, 1, 1.22, 1.45] * threshold). As for pitch discrimination, this paradigm started with a practice block, following a block used to determine the individual threshold. Finally, two blocks of the main task with 200 trials each were performed (resulting in 80 repeats per level). As for pitch discrimination, thresholds were determined using three interleaved one-up two-down staircases with multiplicative step-sizes (starting at coherence levels of 0.6, 0.2 and 0.04 respectively). Due to technical problems with the recording of respiration signals in individual blocks, the data from 3 participants had to be excluded (n = 18).

### Emotion discrimination task

In this paradigm, which was modelled based on Zelano et al.^9^, participants had to categorize individual faces as either displaying an angry or disgusted emotional expression. The images (subtending about 8 degrees, presented for 100 ms) were taken from the FACES database^24^, from which we selected 400 middle-ages faces across both genders and the two emotions. Participants performed a brief training block and two blocks of the experiment with 200 trials each (resulting in 200 trials per emotion). The data from all n = 21 participants could be analysed.

### Visual memory task

In this paradigm participants had to remember a set of images showing diverse objects and later had to recognize those shown previously. The paradigm was modelled based on the study by Zelano et al.^9^ and the images were taken from an existing database^25^. During the exposure phase, a total of 164 images (500 ms presentation time) were shown in random order and participants had no other task than to remember these. During the test phase, participants were presented with those 164 previous images and a set of 164 novel images, in pseudo-random order. Participant’s task was to indicate whether the image was previously seen or new, submitting a response was possible during stimulus presentation. The inter-trial intervals for both phases were 2 – 2.5 s. We investigated the respiration data only during the test period. The data from all n = 21 participants could be analysed.

### Sound detection task

This task involved the detection of an acoustic target sound (100 ms sine-wave tone, 1024 Hz) on a white noise background. The target could take one of four levels (signal to noise ratios (SNR), defined as relative root-mean-square amplitudes of tone and white noise) and could either be present or absent. The white noise had a level of 65 dB SPL. The four SNRs were spaced around participant-specific thresholds ([0.3, 0.6, 1.3, 3] * threshold), which were determined in separate blocks. The paradigm started with a practice block, following a block used to determine the individual threshold. Finally, two blocks of the main task with 240 trials each were performed. Individual thresholds were determined using three interleaved one-up two-down staircases with multiplicative step-sizes (starting at 0.5, 0.1 and 0.02 respectively). Due to technical problems with the recording of respiration signals data from only n = 20 participants could be analysed.

### Data analysis of respiratory data

**Figure 1:**
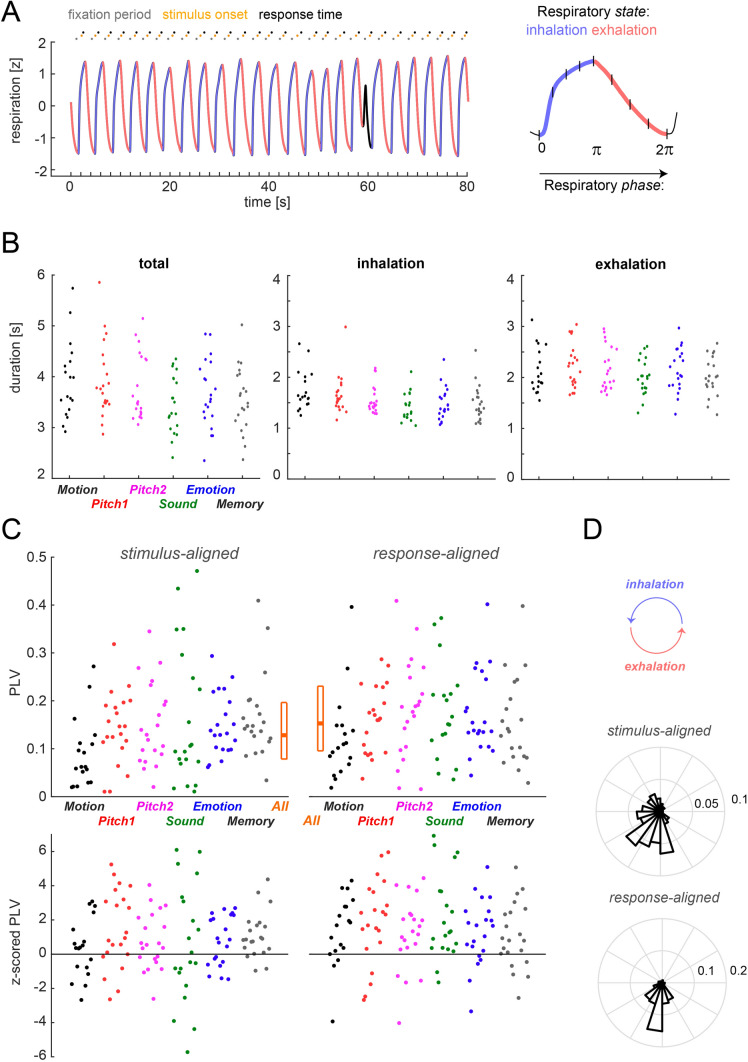
Detection of respiratory cycles and their alignment to paradigm events. (**A**) Respiratory cycles were automatically segmented into inhalation (blue) and exhalation (red) periods, while atypical cycles were excluded (black). The trace shows one example signal (z-scored) together with the timing of trials for one participant in the Sound paradigm. The right panel shows how respiratory variables were coded as either a continuous cyclic variable (to test for an alignment of respiration to the paradigm); or using 4 phase-bins during inhalation and exhalation respectively (to test for a relation between respiration and performance). (**B**) Duration of respiratory cycles, and the inspiration and expiration states separately per paradigm (color-coded) and participant (dots indicate the participant-wise median values). (**C**) Alignment of respiratory cycles to paradigm events (stimulus onset, response times). The upper panels show participant-wise phase-locking values (PLV), the lower panel the same data but z-scored to participant-specific randomization distributions (based on 2000 randomizations). Here positive values indicate stronger phase-locking than expected by chance. (**D**) Distribution of participant-wise average phase values at stimulus onset or response times (derived as circular mean across trials).

The respiratory signals were filtered using 3-rd order Butterworth filters (high pass at 0.05 Hz, low pass at 8 Hz) and subsequently resampled at 100 Hz using the FieldTrip toolbox^26^. To determine individual respiratory cycles, we implemented two procedures to detect local peaks reflecting the peak inhalation in these traces and found that they yielded very comparable results. One procedure detected local peaks in a low-pass filtered version of the data (1 Hz) that were at least 500 ms apart, while another procedure applied the Hilbert transform to the data, and determined local peaks based on the respective phase variable^27^. These provided highly similar results: the number of detected respiratory cycles differed by only 1.1 ± 0.2% (mean ± across participants, n = 122 across datasets). Individual respiratory cycles were determined based on the data in 7 s windows around each peak (Fig. 1A), whereby peaks were included only if the z-scored trace exceeded a level of z = 0.5^9^. To define the state of respiration for each time point, we proceeded as follows. The inspiration period was defined as the continuous period with positive slope prior to the local peak (whereby interruptions of the positive slope shorter than 350 ms were interpolated). The expiration period was defined as the continuous period with negative slope subsequent to the local peak (again interruptions shorter than 350 ms were interpolated). This definition effectively splits the respiratory cycle into three periods: inhalation, exhalation and short exhale pauses (Noto et al. 2018). These pauses were not considered in the analysis of respiratory states.

To exclude atypical respiratory cycles, we used two criteria. First, we compared the overall time courses of individual respiratory cycles using their mean-squared distances and excluded cycles with a distance larger than 3 standard deviations from the centroid of the participant-specific distribution. We also clustered the durations of individual inspiration and expiration epochs and excluded cycles falling outside 3 standard deviations of this centroid for each participant. These atypical cycles were excluded as they do not reflect the prototypical respiration under investigation here. Across all 122 datasets, we detected a total of 42′539 respiratory cycles, of which 41′863 were retained for analysis (98.4%). Together the excluded cycles and the respiratory pauses amounted to a median of 8.3% of time points during the experimental time (mean ± s.e.m.: 9.0 ± 1% across datasets, n = 122).

**Figure 2:**
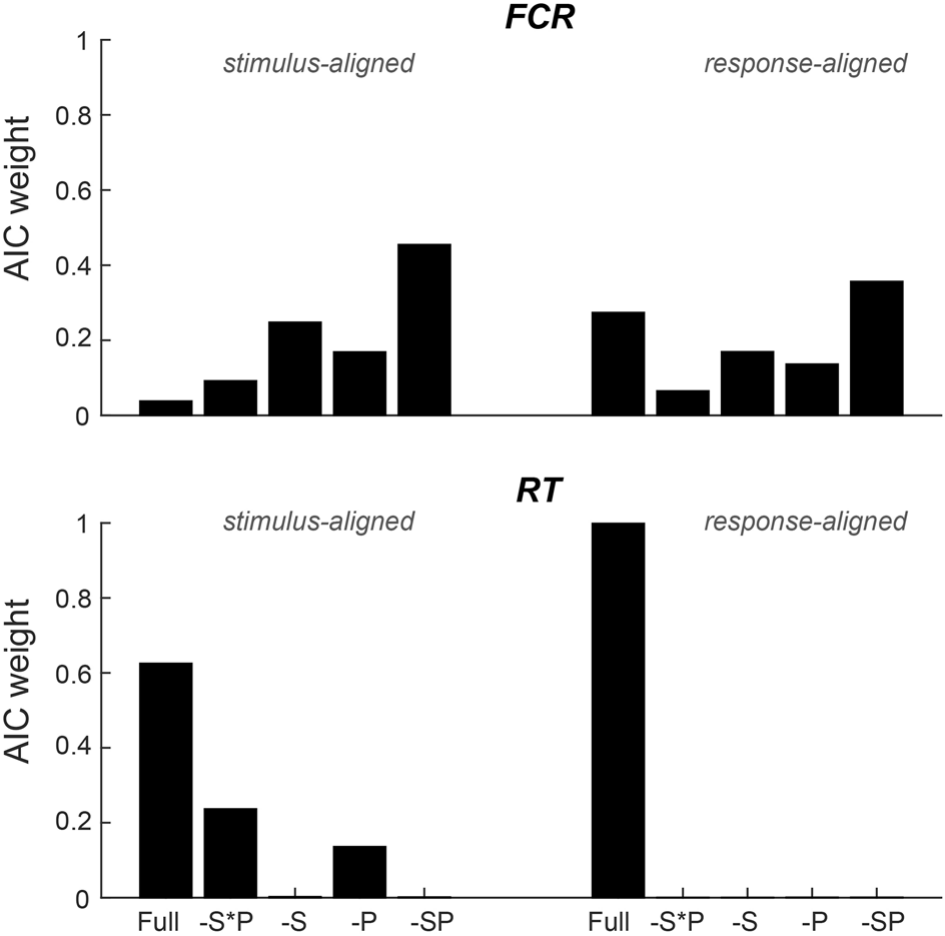
Group-level test for a statistical relation of respiration and behaviour across paradigms. Bars indicate the Akaike information criterion (AIC)-weights for mixed linear models predicting the fraction of correct responses (FCR) or response times (RT) based on all respiratory predictors (full), a model excluding the interaction of respiratory state and respiratory phase (-S*P; c.f. Figure 1A for the definition of state and phase), models excluding the interaction and either state (-S) or phase (-P), and a model excluding all respiratory predictors (-SP). Models were fit separately based on the respiratory variables at stimulus onset or at response times and were fit across all six paradigms and n = 122 datasets (see Table 1 for model parameters).

**Figure 3:**
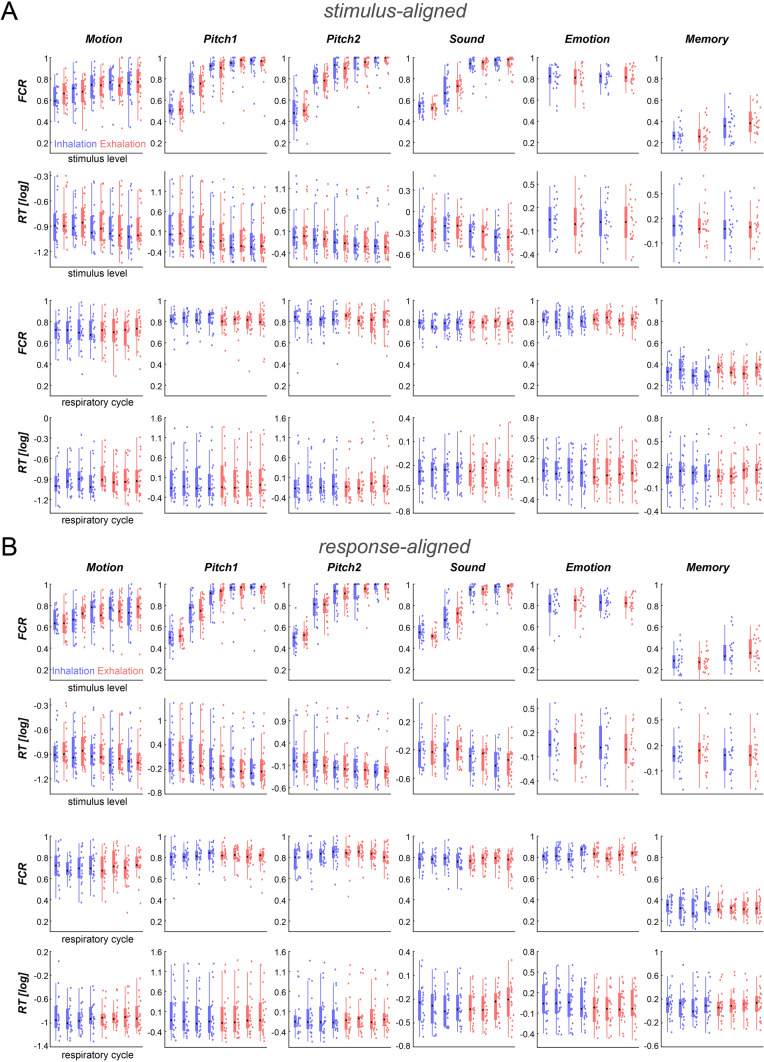
Behavioural performance for individual paradigms and participants. For each paradigm the panels show the fraction of correct response (FCR) or log-transformed reaction times (RT). The upper two panels show these as a function of respiratory state (inhalation, exhalation; color-coded) and stimulus level; the lower two panels as a function of respiratory state and phase bin (averaged over levels). (**A**) Shows the data based on the respiratory variables at stimulus onset. (**B**) Shows the data based on the respiratory variables at response times. Boxplots indicate the central quartiles and median (black circle), individual coloured-dots the participant-wise data.

**Figure 4:**
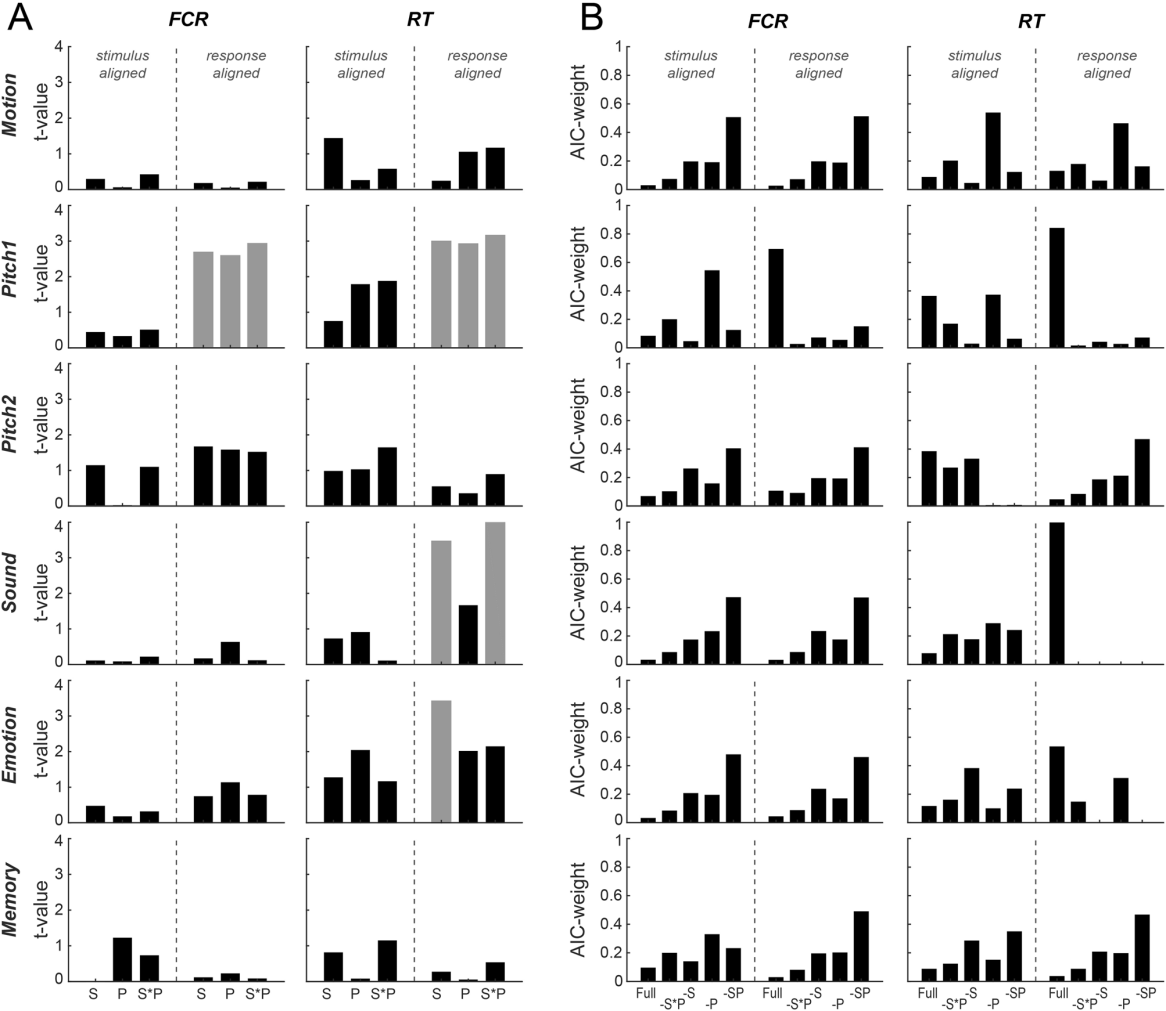
Paradigm-wise mixed-linear modelling of behaviour against respiratory variables. (**A**) Statistical significance (t-value) of model coefficients for linear models fit for individual paradigms modelling the fraction of correct responses (FCR) or response times (RT) based on the respiratory variables at stimulus onset or at response times. Significant predictors are indicated in grey (*p* < 0.01; two-sided t-test, uncorrected). Model predictors were respiratory state (S; inhalation or exhalation), respiratory phase within each state (P) and their interaction (S*P). (**B**) Model comparison based on Akaike information criterion (AIC)-weights. Here the full model including all respiratory predictors (full) was compared to a model excluding the interaction of respiratory state and respiratory phase (-S*P), models excluding the interaction and state (-S) or phase (-P) and a model excluding all respiratory predictions (-SP).

**Figure 5:**
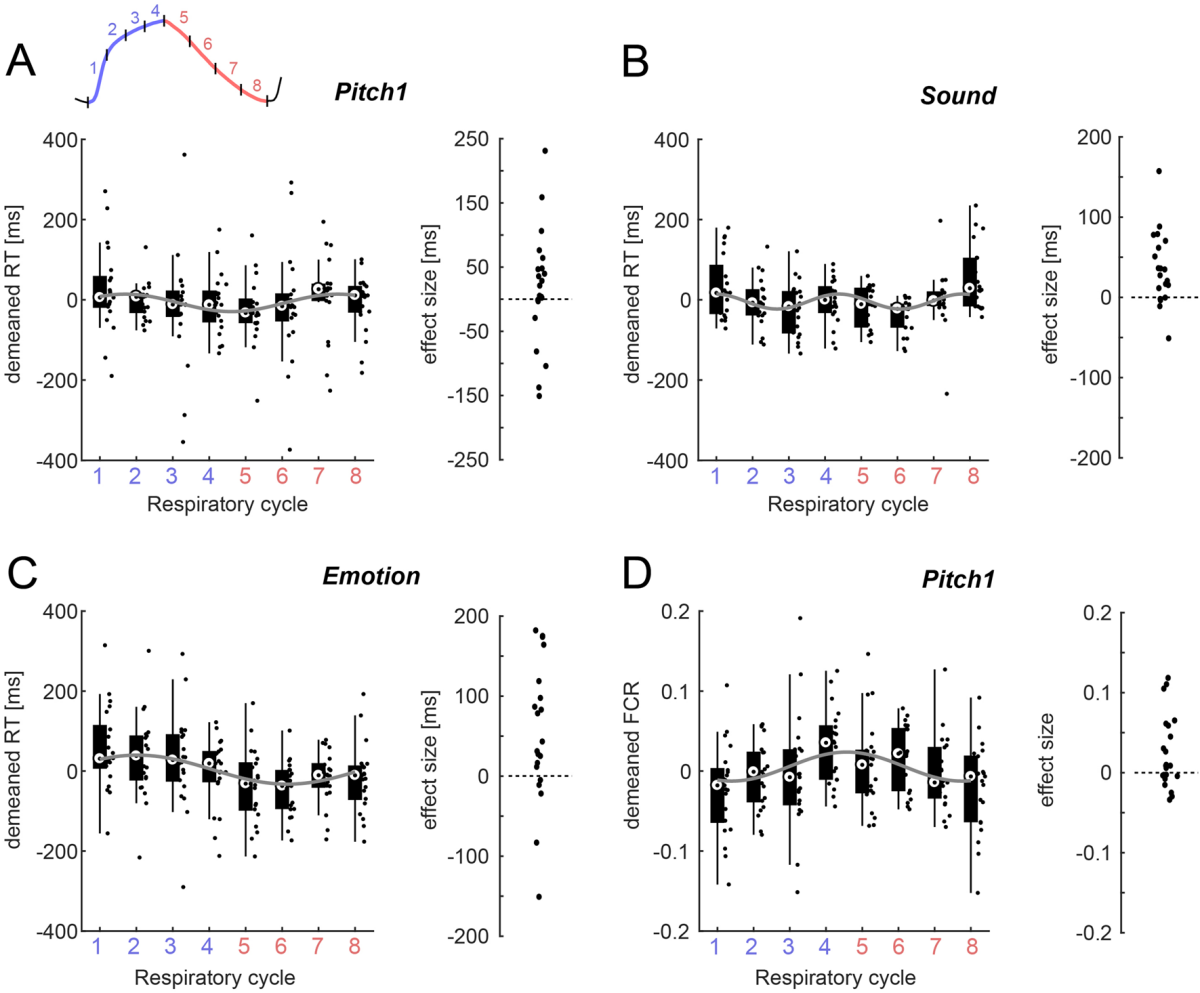
Estimates of effect sizes. For those paradigms with significant effects in linear models (c.f. Table 2) the figure shows the respective variation in reaction times (**A-C**) or the fraction of correct responses (**D**) along the respiratory cycle (inset in Panel A). For display purpose, the individual participant data were mean-subtracted. The group-level median data were fit using a rhythmic model (grey line), based on which we determined those bins with positive and negative RT (or FC). We then derived a measure of effect size as the participant-wise difference in the data between positive and negative bins. This procedure ensures that effect sizes are derived under the assumption of a fixed respiratory pattern across participants within each individual paradigm. The right-hand panels indicate the participant-wise (dots) effect sizes.

To link respiratory signals to behaviour, we relied on the division of respiratory cycles into the two prominent ‘states’ of inspiration and expiration. For each event of interest (stimulus onset, participant’s response times), we assigned the state as the one which prevailed in a 100 ms window around the event. In addition, we subdivided each state into a continuous ‘phase’ variable (Fig. 1A, right panel). The phase was defined as linearly increasing from the beginning to the end of each state. To analyse the relation between respiration and performance this phase was binned into four equally long phase-bins (results in Figs. 2, 3, 4, 5). To probe the alignment of the respiratory cycles to paradigm events (Fig. 1C, D), we coded the phase during inhalation (exhalation) periods as progressing from 0 to pi (pi to 2*pi), so that the full respiratory cycle could be described by a cyclic variable.

### Statistical analysis of the alignment respiration and paradigm

To probe whether participants aligned their respiratory behaviour to the experimental paradigms we proceeded in two ways. First, we coded individual respiratory cycles using their cyclic phase variable and quantified the consistency of this phase across trials using the mean-resulting vector length (phase-locking value PLV). This was done for each participant and event of interest separately (stimulus onset, response time; Fig. 1C, upper panels). For comparison, we derived a surrogate distribution of phase-locking values under the null hypothesis of no alignment between respiratory trace and paradigm for each participant. This was obtained by randomly time-shifting the respiratory trace and recalculating the phase consistency 2000 times. We then z-scored, for each participant and event, the actual PLV against the randomization distribution (Fig. 1C lower panels). To test the hypothesis that participants aligned their respiratory cycle to the paradigm more than expected based on the surrogate distribution, we contrasted these z-scored PLV values against zero using sign-tests (one-tailed, using the approximate method^28^. When testing individual paradigms, we further corrected across the 12 tests (6 paradigms, 2 alignments) using the Bejnamini & Hochberg FDR procedure^29^. We also compared the PLV between stimulus and response-aligned data using a Wilcoxon sign-rank test to probe whether the alignment strength differed between these events. In a second analysis, we probed the hypothesis that across participants the trial-averaged respiratory phase values were not distributed uniformly across the cycle (Fig. 1D). This was tested using Rayleigh's test for non-uniformity, using the circular toolbox in Matlab^30^. To further understand whether participants specifically tended to inhale (or exhale) at the events of interest, we calculated the fraction of trials at which the respiratory state was inhalation for each event. This estimate is generally biased towards exhalation, as the overall duration of exhalation periods was longer. We again z-scored these estimates against a surrogate distribution, obtaining z-values that indicate whether individual participants tended to inhale more frequency than expected by chance given the data.

### Statistical analysis relating respiratory and behavioural variables

To relate respiration and behavioural data, we first removed trials based on either atypical behavioural or respiratory data. Outliers were defined based on reaction times (RTs) being shorter than 200 ms or longer than 3 standard deviations above the mean (based on log-transformed RTs). This led to the exclusion of (median) 1% of trials across datasets (mean ± s.e.m: 3.5 ± 0.8 trials; n = 122). In addition, we removed trials for which the state of respiration was not defined at the events of interest. Collectively these criteria led to the exclusion of a (median) 6.5% and 7.1% of trials for stimulus- and response-aligned data (mean ± s.e.m: 9.7 ± 0.9% and 10.1 ± 0.9% of trials, n = 122). Subsequent analyses linking respiratory data to behaviour focused on log-transformed reaction times (RTs; see below for rationale) and the fraction of correct responses (FCR) per condition of interest and were repeated once using the respiratory variables at stimulus onset and once at the time of participant’s response.

To code respiratory variables for data analysis, we relied on two features: the state of respiration (inhalation, exhalation) and the phase-bin within each state (4-levels). This coding was used to be able to differentiate inhalation from exhalation in the statistical testing, which would not be possible when focusing on a single continuous respiratory phase value. In addition, this coding normalized the overall prominence of inhalation and exhalation periods, which is important given that exhalation periods are generally longer (Fig. 1B). For each paradigm and participant, we grouped trials per stimulus level, respiratory state, and phase. For each of these cells (level x state x phase) we derived the participant-averaged RT and FCR. The factor stimulus level was either the parametrically manipulated saliency of the sensory information (Motion coherence, Pitch differences, Sound signal to noise ratio), the emotion category or object novelty (Memory task). The resulting behavioural data are shown in Fig. 3, averaged across phase-bins (top) or across stimulus levels (bottom). We used mixed linear models to probe whether the behavioural data indeed varied with these factors (level, respiratory state, respiratory phase, and state x phase interaction). Models were fit with participants as random effects, respiratory state as categorical effect and level and phase as linear predictors (fitlme in Matlab; using the ‘quasinewton’ method). Separate models were fit for stimulus or response aligned data. In one approach we fit models separately for each paradigm, in another approach we fit one model across all six paradigms (treating level as paradigm-specific effects). These two analyses probe whether the influence of respiratory variables i) is consistent across participants within a paradigm, or ii) is consistent across participants and also paradigms. As model outcomes we report the predictor coefficients and their 95% confidence intervals, t- and p-values (Tables 1, 2, Fig. 4).

**Table 1:** Relevance of respiratory predictors across paradigms.

	Beta [CI]	t-value	*P *value
**Fraction correct responses—stimulus aligned**			
State	0.007 [− 0.020, 0.034]	0.487	0.6262
Phase	− 0.001 [− 0.008, 0.006]	− 0.288	0.7733
State*Phase	− 0.002 [− 0.012, 0.008]	− 0.479	0.6323
Intercept	0.749 [0.647, 0.851]	14.341	0.0000
LR-Test		Chi-sq(3) = 1.0288	0.79428
**Fraction correct responses—response aligned**			
State	0.029 [0.002, 0.056]	2.133	**0.0330**
Phase	0.007 [0.000, 0.014]	2.071	**0.0330**
State*Phase	− 0.011 [− 0.021, − 0.001]	− 2.211	**0.0271**
Intercept	0.743 [0.642, 0.843]	14.527	0.0000
LR-Test		Chi-sq(3) = 5.4721	0.14032
**Reaction times—stimulus aligned**			
State	− 0.006 [− 0.033, 0.021]	− 0.420	0.6742
Phase	− 0.001 [− 0.008, 0.006]	− 0.157	0.8753
Phase*Phase	0.010 [0.000, 0.020]	1.987	**0.0470**
Intercept	− 0.408 [− 0.668, − 0.148]	− 3.075	0.0021
LR-Test		Chi-sq(3) = 18.617	**0.0003**
**Reaction times—response aligned**			
State	− 0.053 [− 0.081, − 0.025]	− 3.708	**0.0002**
Phase	− 0.011 [− 0.018, − 0.004]	**− 3.015**	**0.0026**
State*Phase	0.023 [0.013, 0.034]	4.471	** < 10**^**–4**^
Intercept	− 0.293 [− 0.559, − 0.026]	− 2.155	0.0312
LR-Test		Chi-sq(3) = 20.818	**0.0001**

The relation between respiration and behavioural performance (Fraction correct responses, log-transformed reaction times) was probed using mixed linear models, which were fit separately using the state and phase of respiration obtained at stimulus onset or the response time (c.f. Figure 1A). The table provides the predictor coefficients (incl. 95% confidence intervals), the respective t- and p-values and the result of a likelihood-ratio test comparing a model with and a model without the respiratory predictors.

Significant values are in bold.

**Table 2:** Relevance of respiratory predictors for individual paradigms.

	Beta [CI]	t-value	p-value
**Pitch 1—FCR response aligned**			
Level	0.102 [0.095, 0.110]	27.467	** < 10**^**–4**^
State	0.069 [0.019, 0.120]	2.685	**0.0074**
Phase	0.017 [0.004, 0.030]	2.589	**0.0098**
State*Phase	− 0.028 [− 0.046, − 0.009]	− 2.927	**0.0035**
Intercept	0.453 [0.400, 0.505]	17.033	< 10^–4^
**Pitch 1—RT response aligned**			
Level	− 0.065 [− 0.073, − 0.057]	− 15.917	** < 10**^**–4**^
State	− 0.085 [− 0.141, − 0.029]	− 2.995	**0.0028**
Phase	− 0.021 [− 0.036, − 0.007]	− 2.921	**0.0036**
State*Phase	0.033 [0.012, 0.053]	3.158	**0.0016**
Intercept	0.266 [0.038, 0.493]	2.288	0.0224
**Sound—RT response aligned**			
Level	− 0.060 [− 0.070, − 0.050]	− 12.196	** < 10**^**–4**^
State	− 0.093 [− 0.146, − 0.040]	− 3.465	**0.0006**
Phase	− 0.012 [− 0.025, 0.002]	− 1.666	0.0962
State*Phase	0.041 [0.022, 0.061]	4.194	** < 10**^**–4**^
Intercept	− 0.107 [− 0.207, − 0.008]	− 2.117	0.0346
**Emotion—RT**			
Level	− 0.008 [− 0.034, 0.019]	− 0.571	0.5682
State	− 0.112 [− 0.177, − 0.048]	− 3.417	**0.0007**
Phase	− 0.017 [− 0.034, − 0.000]	− 2.019	**0.0443**
State*Phase	0.026 [0.002, 0.049]	2.147	**0.0325**
Intercept	0.094 [− 0.026, 0.214]	1.546	0.1231

The table lists liner model results for those paradigms and alignments that returned a significant effect of respiratory variables. The results (t-statistics) for all paradigms, alignments and variables are shown in Fig. 4.

Significant values are in bold.

To further substantiate whether the addition of the respiratory predictors improved the model fit, we contrasted models with and without the respiratory predictors using likelihood ratio tests (Table 1). Furthermore, we systematically contrasted the explanatory power of models including or excluding individual respiratory predictors based on their respective log-likelihoods: we fit models including all three respiratory predictors, a model excluding their interaction, two models excluding the interaction and either state or phase, and a model excluding all three respiratory predictors. We used the Akaike information criterion (AIC) to derive the conditional probability of each model given the data using the Akaike weights^31^.

We based the analysis of reaction times on the log-transformed data as a preliminary analysis had revealed that this allowed the best description of the expected effects of the stimulus manipulations (levels). Specifically, for the four tasks with expected effects of stimulus levels (Motion, Pitch 1&2, Sound) we applied the above described linear mixed models to only the factor level, after deriving the level-specific average reaction time using three different coding schemes^32^: the raw data, the log-transformed data, or the inverse-transform. This revealed that the log-transformed data allowed the best linear-model-based description based on the summed AIC across all four paradigms (AICs for raw values, log- and inverse-transform: − 306, − 526, − 351), although all three coding schemes allowed capturing the effect of level well (adjusted R^2^: 0.96, 0.96, 0.95).

### Analysis of effect sizes

To quantify effect sizes for the dependency of the behavioural data on respiratory variables (Fig. 5), we first described the group-level median data using a rhythmic dependence on respiratory time, after averaging out the factor level. For this we compared the explanatory power (log-evidence) of models involving distinct timescales between 0.5 and 3 Hz per respiratory cycle, and derived the best-fitting time-scale (Fig. 5; grey curves). We then used these rhythmic descriptors to quantify an effect-size of how much reaction times or the FCR vary along the respiratory cycle: for each paradigm and factor of interest we computed the difference in reaction times (or FCR) between the bins with positive or negative rhythmic components. Note that this fitting of rhythmic models was only used to decide on which phase-bins to contrast, not to substantiate the statistical significance of a rhythmic modulation. These effect-sizes were derived for each participant individually and reflect the expected modulation of RT (of FCR) under the assumption that the time course of such modulation is the same across participants per paradigm. Group-level effect sizes were quantified using their mean, median and Hedges’g. The 95% confidence intervals for the mean were obtained from the respective t-distribution, for the median they were obtained using the Harrell-Davis estimator based on the ‘matrogme’ Matlab package^33,34^, and for Hedges’g they were obtained using bootstrapping using the Effect size toolbox in Matlab based on 2000 bootstrap samples^35^.

## Results

### Properties of respiratory cycles

We collected behavioural data during six paradigms involving the detection or discrimination of either visual or acoustic stimuli or testing participant’s memory of these. This resulted in a total of 122 datasets. During each of these experiments participant’s respiration was recorded using a thermistor inserted into a face mask. This provided high resolution traces reflecting the temperature changes induced by inhalation and exhalation (Fig. 1A). These were automatically segmented into individual respiratory cycles. Across datasets we detected a total of 42′539 cycles, of which 98.4% were retained for analysis. The time course of individual cycles was split into two variables for analysis: the respiratory state was defined as either inhalation or exhalation (Fig. 1A; color-coded); the time within each state was divided into a phase variable, which increased linearly from the start to the end of each state (Fig. 1A; right panel).

Across participants the median duration of respiratory cycles was 3.57 s ([25th, 75th percentiles]: [3.25, 4.15] s, n = 122; durations were averaged within participants across detected cycles, Fig. 1B), corresponding to a respiratory frequency of ~ 0.3 Hz. As expected, inhalation states were shorter than exhalation states (inhalation: 1.49 s [1.34, 1.69] s; exhalation: 2.03 s [1.82, 2.41] s). Figure 1B provides the respective durations per participant and paradigm.

### Respiratory cycles are aligned to paradigm events

Previous studies suggested that participants may potentially adjust their respiratory behaviour to an experimental paradigm, for example by aligning their respiratory cycle with expected events such as stimulus presentation or their responses^7^. We hence probed for signatures of such behavioural strategies in the present data. In the paradigms tested here, the stimuli were presented at pseudo-random intervals following a fixation onset (0.4—1 s uniformly distributed) or following participants self-initiated trial-start (Pitch 2, 0.3—0.6 s, uniform delay). Alternatively, participants may have aligned their respiratory pattern to their responses.

We tested for such an alignment by computing for each participant the phase-locking value (PLV) characterizing the consistency of the respiratory cycle across trials (Fig. 1C, upper panels). We then compared the resulting PLV within participants to a surrogate values obtained under the null hypothesis of no alignment between respiratory trace and the experimental paradigm (Fig. 1C, lower panels). We first tested the hypothesis that the alignment in the actual data was stronger than in the surrogate data: across paradigms this was the case both for stimulus onset and at participant’s response times (n = 122; one-sided sign-tests, stimulus onset Z_sign-test_ = 3.5, p = 0.004, response time Z_sign-test_ = 6.8, *p* < 10^–4^). Repeating this test for individual paradigms returned a significant effect for each paradigm in the response-aligned data (corrected using the Bejnamini & Hochberg FDR procedure across the 12 tests, minimal Z_sign-test_ = 2.1, at least p_corr_ < 0.02) and for the memory paradigm also in the stimulus-aligned data (Z_sign-test_ = 3.0, p_corr_ = 0.004).

Inspecting the individual participant data revealed that for many participants the observed PLV values were significantly stronger than expected based on their individual surrogate data (individual z scored PLVs larger than z = 2.33, which corresponds to a one-sided 99% critical level: 30 of 122 for stimulus onset and 41 of 122 for response time; Fig. 1C, lower panel). This difference between stimulus and response-aligned data seems to suggest that the alignment was stronger when analysed contingent on response time. Indeed, a comparison of the actual PLVs between stimulus and response aligned data revealed a significant difference (Wilcoxon sign-rank test, Z_sign-rank_ =  − 4.2, *p* < 10^–4^): the PLV values were higher at response time (median and [25th, 75th percentiles] for response time: 0.13 [0.08, 0.2] and for stimulus onset: 0.15 [0.1, 0.23]; Fig. 1C, upper panel). Because paradigms pitch 1 and pitch 2 differed only in that participants actively initialized a trial (pitch 2) rather than this being under pseudo-random control (pitch 1) we directly contrasted the PLV values between these: this did not reveal any significant difference (stimulus-aligned: Z_sign-rank_ =  − 0.02, p = 0.82; response-aligned: Z_sign-rank_ =  − 1.44, p = 0.15).

To understand how precisely respiratory cycles were aligned to the paradigm we investigated the trial-averaged respiratory phase angles (coded as 0 to pi for the inhalation period and pi to 2 pi for exhalation Fig. 1D). The average phase angles were highly clustered across participants and deviated significantly from a null hypothesis of a uniform distribution (Rayleigh tests, Z_Rayleigh_ = 25 and Z_Rayleigh_ = 72, both *p* < 10^–4^ for stimulus and response-aligned data). These data visualize the stronger alignment of respiratory behaviour around response time across participants and support the conclusion that participants systematically aligned their respiratory behaviour to the paradigm. However, because exhalation periods were generally longer, the average phase angle in Fig. 1D may confound the prominence of exhalation periods with an alignment specifically emphasizing exhalation around response times. In a final analysis we hence calculated the fraction of trials featuring an inhalation state at the events of interest, and z-scored this within participants against the individual expected proportion based on surrogate data. We then asked whether the median proportion of excess (vs. surrogate) inhalation states deviated from zero. Across datasets, this revealed a significant shift towards inhalation near stimulus onset (median z = 0.64, Wilcoxon sign-rank test, Z_sign-rank_ = 3.2, p = 0.001) and a shift towards exhalation near response times (median z =  − 0.47, Z_sign-rank_ =  − 0.32, p = 0.001).

### Behavioural data covary with respiration

We then asked whether participants behavioural performance revealed evidence for a systematic variation along the respiratory cycle. For each paradigm, we focused on the fraction of correct responses (FCR) and participant’s reaction times (RTs) and related these to the respiratory variables state and phase. We tested for such relations using linear mixed models, separately for the respiratory variables obtained at stimulus presentation or response times. In a first analysis, we probed for such a relation across all six paradigms, hence testing the hypothesis that behavioural performance varies with respiration consistently across experiments. Across datasets we included (based on acceptable behavioural and respiration data) around 93% of all trials in the analysis (c.f. Material and Methods). We first grouped trials based on the respiratory variables (state, phase, their interaction) and based on stimulus level (depending on paradigm). We then computed for each participant the FCR across trials per group and the trial-average log-transformed RTs. The model was fit across participants (random effects) to predict FCR or RT based on stimulus level (fixed effect per paradigm) and respiration status (fixed effects).

Across the four analyses (FCR and RT; stimulus and response alignment) we found clear evidence that RTs were related to respiration. For RTs, the interaction of respiratory state and phase was a significant predictor in the stimulus-aligned data (*p* < 0.05; see Table 1 for detailed results) and all factors were highly significant for the response-aligned data (*p* < 0.001; see Table 1). To substantiate this observation, we contrasted the GLMMs with and without the respiratory predictors. Likelihood-ratio tests returned a significant improvement in model fits for both alignments when including respiration (*p* < 0.001; see Table 1). In addition, we also compared models featuring only individual respiratory variables and a reduced model excluding all respiratory predictors. For each model we derived its conditional probability within the range of tested models based on Akaike weights (Fig. 2). This yielded clear evidence for an association of respiratory variables and RTs in the response aligned data (AIC-weight for the full model was 0.998) but not the stimulus-aligned data (AIC-weight of full model 0.62). In sum, these results show that participant’s reaction times are associated with the respiratory cycle near the time of these responses.

For the FCR, the respiratory predictors were marginally significant in the response-aligned (*p* < 0.05; see Table 1) but not the stimulus-aligned data. Likelihood-ratio tests returned no evidence for an improvement in model fit when including respiratory predictors (p > 0.05; Table 1) and the comparison of individual models also returned no evidence for an association of FCR and respiration based on AIC-weights (Fig. 2).

### Paradigm specific results

These results point to a systematic relation between respiration and reaction times but not response accuracy. Yet, probing for generic and paradigm-independent effects may also obscure more specific results, such as for example stronger effects for the paradigms involving medial temporal brain structures (Emotion, Memory), for which previous studies make clear predictions^9^.

We hence repeated the analyses for individual paradigms. Figure 3 displays the individual and group-level behavioural data showing the effects of stimulus level and respiratory state (upper two panels in A, B) and showing the effects of the respiratory state and phase averaged across levels (lower two panels in A, B). For each paradigm, we again used linear mixed models to probe the relation between behaviour and respiration. As expected, for each paradigm manipulating stimulus saliency in a parametric fashion (Motion, Pitch1, Pitch 2, Sound), the effect of stimulus level was significant for both FCR and RTs and for both alignments (all t-values > 4.5, *p* < 10^–4^). For the discrimination of emotional faces (Emotion), the effect of emotion was not significant for neither variable (FCR nor RTs) or alignment (all t < 1.2, p > 0.25). For the memory test, the effect of novelty was significant for FCR (both alignments t > 6.1, *p* < 10^–4^) and for RTs (t = 3.9 and 2.5, p = 0.0001 and 0.012 for stimulus and response alignment).

The effects of the respiratory variables are summarized in Fig. 4A, which shows the respective t-statistics and significances for individual predictors (color-coded). Figure 4B shows the conditional probabilities (AIC-weights) for the full model and models excluding individual respiratory predictors. When modelling FCR, respiration was a significant predictor in the response aligned data for Pitch 1 (see Table 2 for details); in this case the full model including all respiratory predictors had the highest probability in this case (AIC-weight 0.69). For all other paradigms and alignments, the model excluding all respiratory predictors generally had the highest conditional probability when modelling FCR (Fig. 4B).

When modelling RTs, the respiratory variables at the response time were significant predictors for three paradigms: pitch discrimination (Pitch1; c.f. Table 2 for details), sound detection (Sound) and the discrimination of emotions (Emotion). For these paradigms the full model including all respiratory predictors also had the highest conditional probabilities (AIC-weights 0.84, 0.99 and 0.53; Fig. 4B). In contrast, the respiratory variables derived at stimulus onset were not significant predictors for RTs in any paradigm (Fig. 4A). Collectively these results confirm a significant relation between respiration at the time of participant’s responses and behaviour performance across a number of paradigms.

One experiment involved the detection (rather than discrimination) of stimuli and was additionally analysed using the framework of signal detection theory. For the Sound paradigm, we calculated hit- and false alarm-rates as well as d’ and bias (n = 22). The linear models for none of these parameters revealed a significant effect for any of the respiratory features (t < 1.1, p > 0.26) and the AIC-weights did not show evidence for models including respiration to perform better than those without (no model exceeded an AIC-weight of 0.5). Hence, we did not find evidence for any change in response behaviour with respiration in this paradigm other than of the reaction times.

### How much does behaviour co-vary with respiration?

We investigated the relation between the respiratory variables and behaviour for those paradigms with significant effects in more detail. Figure 5 visualizes individual participant’s reaction times (in milliseconds) and the fraction of correct responses (only for Pitch 1) along the respiratory cycle. To obtain effect sizes we subtracted RTs (FCR) of those four bins with higher performance from those four with lower performance. To determine those bins under the assumption of a consistent group-level effect, we first derived the best-fitting rhythmic description of the group-level data using a sinusoidal dependence on respiratory time (Fig. 5, grey lines). Based on this rhythmic description we decided on how to group bins to determine effect sizes (see legend Fig. 5).

For comparison with previous work we quantified the effect sizes and their confidence intervals using three descriptors. The group-level mean effect sizes for RTs were 17 ms, 36 ms and 67 ms (95% confidence intervals [− 23, 58]ms, [15, 57]ms, and [18, 114]ms respectively; n = 21, 20, 20). The median effect sizes for RTs were 20 ms, 30 ms, 54 ms (with 95% bootstrap confidence intervals of [− 8, 46]ms, [12, 55]ms, and [17, 109]ms). When quantified using Hedges’ g the effects were 0.19, 0.79 and 0.62 (95% bootstrap confidence intervals [− 0.22, 0.67], [0.42, 1.3], [0.22, 1.14]). The FCR for Pitch 1 varied by a mean of 0.025 ([0.0045, 0.046], n = 21), a median of 0.013 ([− 0.002, 0.04]), with Hedge’s g of 0.55 ([0.19, 0.95]).

## Discussion

We asked whether and by how much participant’s performance in diverse perceptual tasks varies along the respiratory cycle. Across six sensory detections, discrimination, and a memory task we found that participants tended to align their respiratory cycle to the experimental paradigm as shown by the significant phase-locking of the respiratory cycle to the response times. In addition, in several tasks reaction times consistently and significantly varied along the respiratory cycle (pitch discrimination, emotion discrimination and sound detection, but not in a memory, a visual motion discrimination and a second version of the pitch discrimination task), while response accuracy varied little. This covariation of human behaviour with the respiratory cycle was stronger when analysed contingent on the state of respiration around participant’s responses than around stimulus onset. These results support an intricate relation between respiration and sensory-cognitive function and show that the respiratory state may be an important factor to consider when investigating cognition and its neural underpinnings.

### Effect sizes of the respiratory-behaviour coupling

A number of studies have shown that human performance in sensory^10,11^, mental^7,9,12–14^, memory^9,15–17^, or motor tasks^18,19^ varies along the respiratory cycle. Yet, obtaining a comprehensive picture has remained difficult for a number of reasons. Different studies used different technical and statistical approaches to detect variations in behavioural performance along the respiratory cycle, making it difficult to compare effects across experimental tasks. Furthermore, many studies instructed participants to a specific type of respiration, thus possibly biasing attention to their own respiration^6,8,14^. In contrast, we here systematically assessed the relation between task performance and respiration across six tasks in a large participant sample (n = 122) that were performing these laboratory tasks in a typical ‘every-day’ experimental setting and without a specific manipulation of how they were supposed to breath.

The covariation of respiratory cycle and behaviour was more prevalent when analysed based on the respiratory cycle extracted around participant’s responses, both when combining the data across paradigms and when considering them individually. Possibly, this relates to a tendency of participants to align their respiration around their responses rather than to the more uncertain stimulus onset. This view is consistent with a direct and possibly mechanistic relation between respiration and reaction times, as discussed below, although determining any putative causality and its direct neural mechanisms requires further work. We did not find a significant difference in the alignment of the respiratory cycle to paradigm events in a direct comparison of versions of the same task in which the trial-onset was once timed pseudo-randomly (pitch 1) and once linked to participants starting the trial manually (though still with a temporal uncertainty, pitch 2). This does not speak against the general notion that participants tend to align their respiratory cycle with expected paradigm events^7^, but may simply be due to participants already engaging such an active respiratory strategy also in a context with general temporal uncertainty in the stimulus presentation.

While previous studies often focused on detecting statistically significant relations between behaviour and respiration, we also investigated the respective effect sizes and their associated uncertainty. This is particularly important to understand by how much task performance changes along the respiratory cycle. In our data changes in response accuracy were either statistically insignificant (5 out of 6 paradigms) or were small (1.3% of correct responses). Importantly, these effects were associated with confidence intervals including zero and hence do not allow a firm conclusion on a positive effect. This observation is consistent with previous work reporting no significant effect on discrimination performance in the same emotion task as used here^9^, were our data replicate the absence of a significant effect reported previously. Contrasting this, one study reported significant differences in a visuo-spatial shape discrimination task on the order of 5% and replicated in this in two groups of participants. However, the respective confidence intervals reported in that study included zero, hence casting doubts on the existence of a clear effect^7^, experiment 4). Furthermore, the same study found no significant relation to task performance in a lexical decision task^7^. A recent study looking at a visual discrimination task quantified the change in psychometric threshold as a function respiratory cycle and reported shifts on the order of 5% of the respective thresholds, but no confidence for these effects was reported^36^. A notable deviation from this picture comes in an older study investigating the detection of visual signals, which reported a 22% difference in detection rates between inhalation and exhalation periods (95%-Students CI derived from the data reported in that paper: [19–26] %^10^. In sum, we take this body of work to suggest that any relation between response accuracy and respiration is small and most likely not consistent across sensory-cognitive domains.

Related to this, we note that our replication of the memory task used in a previous study did not reveal a significant relation between memory recall performance and respiration. This is in contrast to the previous study, which had reported an accuracy difference of about 5% between inhalation and exhalation (estimated Students CI derived from the data reported in that paper: [1.1–8.8] %). While we cannot directly explain this difference, we note that the effect reported previously was derived from a smaller participant sample and hence is associated with a larger statistical uncertainty. Hence, whether and to what degree memory recall is related to respiration behaviour remains to be tested more carefully in the future.

The visual motion discrimination task, which was based on the classical paradigm of random dot kinetograms, did not yield any significant results. Whether the lack of respiration-related effects in this visual task, in contrast to the two auditory paradigms, is linked to the sensory modality remains unclear. Some previous studies have pointed to respiration-related changes in visual task performance^7,36^, but possibly the duration of the respective stimuli (340 ms for the visual task, and 150 ms or shorter for the auditory tasks) also contributes to these apparent differences between sensory modalities.

In contrast to task performance, reaction times covaried significantly with the respiratory cycle in 3 out of 6 paradigms tested here. Based on the associated confidence intervals, the effects sizes for RTs fall in between 10 to 110 ms, with median values between 20 and 50 ms. This order of effect is consistent with previous studies reporting a change in RTs for a sound detection task with a mean effect size of 17 ms for spontaneous breathing and 61 ms for controlled breathing^11^,confidence interval could not be extracted as no data variability was reported). Similarly, a study on the discrimination of emotional faces reported an effect size of 16 ms (95% CI estimated from the reported data: [4–29]ms;^9^ Emotion task with nasal breathing). In some studies, even stronger effects were found for controlled or deep respiration, as well as forced oral respiration, suggesting that attention to respiration can further enhance these effects^9,11,37^. Together these studies suggest that reaction times during sensory-cognitive tasks indeed vary systematically with the state of respiration.

To put the effect size of changes in reaction times along the respiratory cycle in context, we here compare these to other and well-established changes in reaction times in sensory-cognitive paradigms. For example, a meta-study on the effect of transcranial magnetic stimulation in attention and memory tasks^38^ reported an overall effect size of 0.24 (Hedge’s g,95% confidence interval [0.05, 0.42]). Similarly, a meta-analysis of the effects of transcranial direct current stimulation on inhibitory control reported even smaller effect sizes, with an average of 0.10 (Hedge’s g; 95% confidence interval [0.02, 0.21] and a largest effect size per individual study of up to 0.83^39^. Compared to such effects of brain stimulation, the effect sizes of respiratory-related changes in reaction times are very comparable (Hedge’s g between 0.19 and 0.79). Comparable effect sizes were also reported for cognitive interference effects in clinical populations (average of Cohen’s d of 0.67 in^40^) and in a larger meta-analysis of a large body of cognitive neuroscience and psychology literature (average d of 0.66^40^). Hence, changes in reaction times along the respiratory cycle may be well comparable to other changes in reaction times induced by typical sensory-cognitive paradigms.

### Respiration as potentially confounding factor in sensory-cognitive studies

These results also suggest that respiration may act as a confounding factor in sensory-cognitive paradigms, in particular as participants may align their respiratory pattern to specific experimental manipulations. Neuroimaging studies have considered respiration as a major confounding factor for a long time, via the associated movements of body and brain^41,42^. However, respiration may also affect heartrate^43^ and thereby neuroimaging signals indirectly: the visibility of ECG-related signals in electro-magnetic brain measurements and pulsation-related effects can distort these in a respiration-specific manner. The sensation of one owns heartbeat is reflected in neural activity in the brain^44,45^, hence leaving a route for respiration to affect brain activity also via other interoceptive mechanisms. Collectively, this suggests that respiration may not only be a potential confounder for measurements of brain activity but may also shape, directly or indirectly, brain activity and behaviour^6,46^. One could imagine that such respiration-mediated effects go hand in hand with other effects by which the brain predicts upcoming events in an experimental paradigm, or their perceptual salience, as these can provide reliable cues for respiration to align with.

### Potential causal roles of respiration

Measurements of brain activity have shown that brain activity aligns to the respiratory cycle not only within olfactory structures, but also in brain regions connected with the olfactory system such as the limbic system^47^, and even in more distant regions such as parietal or prefrontal cortex^3–5,8,48,49^. While the specific impact of respiratory signals in higher association regions remains to be fully understood, recent studies have shown that such respiratory-modulations also extend into the motor system, offering one of potentially several pathways by which reaction times may be altered in a respiratory-specific manner^3,19^.

A second pathway for respiration to shape perception may be via the modulation of neural excitability in sensory regions. An MEG study showed that parieto-occipital alpha band activity modulates along the respiratory cycle^49^. In that study the significant respiration-alpha coupling temporally preceded performance changes in perceptual accuracy that were also related to respiration. Alpha band activity reflects the excitability of cortical circuits^50–52^ and is an omnipresent predictor of perceptual performance, reaction times or metacognition in sensory-cognitive tasks^53–55^. In particular, studies have shown a correlation between alpha power and more direct measures of neural activity^6,52,56^. If respiration indeed shapes alpha band activity^57^, it is not surprising hence, that respiration shapes perceptual performance across a range of tasks. Given that alpha band activity is also known to be influenced by factors such as spatial or cross-modal attention^58,59^, temporal expectation^60^ or idiosyncratic biases^61^, it remains unclear to what degree any respiratory influence becomes visible in each individual experimental paradigm. Our comprehensive analysis across six tasks suggests that effects on response speed may be more prevalent than effects on task performance.

In this context it is important to note that previous studies directly comparing the nasal and oral routes of respiration point to stronger effects for nasal breathing, or when breathing is under explicit voluntary control^11,37^. For example, the respiratory modulation of behaviour during an emotion discrimination task and the modulation of neural oscillations in medial temporal regions were significantly stronger during nasal compared to oral breathing^9^. However, another study using a visuo-spatial task found a behavioural modulation by both oral and nasal breathing^7^. In the present study we asked participants to breathe through their nose as usual, but we cannot rule out that oral breathing was performed at least during parts of the experiments. Thereby our results approximate the ‘typical’ effect likely to be present in experimental settings devoid of an explicit focus on respiration and suggest that during such natural conditions variations in reaction times with respiration may be rather widespread.

## Conclusion

The cerebral networks receiving external sensory information and those related to interoception are intricately linked^62^, and studies show that the homeostatic state and bodily functions such as heartbeat or respiration bear an influence on perception. For example, the endocrine system can shape perceptual thresholds via levels of glucocorticoids^63^, while the heartbeat affects perception not only in the somatosensory domain^64^ but also influences value-based decisions^65^ and can guide visual exploration search^66^. In line with this, a growing body of results shows that performance during sensory or cognitive tasks is linked to the nature and state of respiration^3,7,9,36,67^. Our data support this view and show that behavioural performance in typical sensory tasks can be systematically structured relative to the respiratory cycle.
